# The characteristics of familial precocious puberty

**DOI:** 10.1186/1687-9856-2015-S1-P97

**Published:** 2015-04-28

**Authors:** Hwal Rim Jeong, Eun Byul Kwon, Young Seok Shim, Hae Sang Lee, Jin Soon Hwang

**Affiliations:** 1 Department of Pediatrics Ajou University, School of Medicine, Suwon, Korea

## Aims

Precocious puberty is defined as the precocious onset of pubertal manifestations. The cause of precocious puberty is unknown despite numerous attempts to find it. Despite most precocious puberty is sporadic disease, some patients have familial tendency. Recently specific gene mutation has proven to cause precocious puberty and the existence of familial precocious puberty is emerging. This study was performed to compare the characteristics of familial precocious puberty and sporadic precocious puberty.

## Methods

We studied 32 girls diagnosed with central precocious puberty (CPP) at Ajou University Hospital from 1st Jan 2007 to 31th May 2014. 16 girls have sisters diagnosed with CPP and the other 16 children have no family history of CPP. We divided these subjects to two groups, familial CPP and sporadic CPP. All subjects had been treated with GnRH agonist. We reviewed their auxological data, Tanner stage, laboratory findings, and bone age retrospectively.

## Results

The onset of precocious puberty was not available. Baseline characteristics including mid parental height (MPH), bone age, height SD, the bone age advancement, body mass index, Tanner stage and LH peak on GnRH stimulation test revealed no significant difference between familial CPP and sporadic CPP. Age at diagnosis (yr) was 8.54 ± 0.48 and 8.11 ± 0.68 respectively (p = 0.049). The GnRH agonist treatment period (yr) was 3.26± 0.39 and 2.96 ± 0.79 respectively (p > 0.05). The growth velocity (cm / yr) during the treatment was 5.36 ± 0.55 and 5.34 ± 0.85 respectively (p > 0.05). After the GnRH agonist treatment was finished, the increment of PAH (cm) was 12.14 ± 4.42 and 10.83 ± 4.98 respectively (p > 0.05).The difference between PAH and MPH was 6.52 ± 4.92(cm) (p < 0.01) in familial CPP and 1.13 ± 4.18 in sporadic CPP (p > 0.05).

## Conclusion

The clinical manifestation of familial precocious puberty is similar with sporadic precocious puberty. GnRH agonist treatment increases the predicted adult height above the mid parental height in familial precocious puberty.

